# Chemical Composition and Antioxidant Activity of Essential Oil of Six *Pinus* Taxa Native to China

**DOI:** 10.3390/molecules20059380

**Published:** 2015-05-21

**Authors:** Qing Xie, Zhihong Liu, Zhouqi Li

**Affiliations:** College of Forestry, Northwest A & F University, Yangling 712100, China; E-Mails: qingxie183@163.com (Q.X.); liuhong0543@163.com (Z.L.)

**Keywords:** pine needle, essential oil, GC/MS, antioxidant activity

## Abstract

The essential oils obtained by steam distillation from needles of six China endemic *Pinus* taxa (*P*. *tabulaeformis*, *P*. *tabulaeformis* f. *shekanensis*, *P*. *tabulaeformis* var. *mukdensis*, *P*. *tabulaeformis* var. *umbraculifera*, *P*. *henryi* and *P*. *massoniana*) were analysed by GC/MS. A total of 72 components were separated and identified by GC/MS from the six taxa. The major constituents of the essential oils were: α-pinene (6.78%–20.55%), bornyl acetale (3.32%–12.71%), β-caryophellene (18.26%–26.31%), α-guaiene (1.23%–8.19%), and germacrene D (1.26%–9.93%). Moreover, the essential oils were evaluated for antioxidant potential by three assays (DPPH, FRAP and ABTS) and tested for their total phenolic content. The results showed that all essential oils exhibited acceptable antioxidant activities and these strongly suggest that these pine needles may serve as a potential source of natural antioxidants for food and medical purposes.

## 1. Introduction

Oxidative stress reflects an imbalance between the systemic manifestation of reactive oxygen species and their elimination by protective mechanisms, referred to as antioxidants [1]. Because of their oxidant properties, these species can damage all components of the cell, including proteins, lipids, and DNA [2] and consequently leading to cell injury and the development of various physiological and pathological abnormalities such as aging, neurodegenerative diseases (Alzheimer’s, Parkinson’s and Huntington’s) [3], cardiovascular diseases (atherosclerosis, heart failure) and many cancers [4]. Interest in extracts and biologically active compounds isolated from popular plant species has recently increased. Large number of plants worldwide has been investigated for their antioxidant activity [5,6,7,8]. This antioxidant capacity can be explored in food industry to maintain food quality by using plants as a source of antioxidants to prevent the rancidity and oxidation of lipids. Recent years, researches were mainly focused on medicinal plants to extract natural antioxidants that can replace synthetic additives such as butylated hydroxyanisole (BHA) and butylated hydroxytoluene (BHT) that might be toxic and even carcinogenic [9].

The genus *Pinus* belongs to the family Pinaceae and comprises about 115 species. It is the largest genus of conifers occurring naturally in the Northern hemisphere, especially in the Mediterranean region, Caribbean area, Asia, Europe, North and Central American [10]. A total of 22 species and 10 varieties of the genus *Pinus* are distributed in China, and 11 of them are endemic [11]. The needles of the genus *Pinus* are widely used in folk medicine and as food additives due to their numerous pharmacological properties, such as anti-aging and anti-inflammatory effects [12]. The leaves of the genus *Pinus* have been used to prepare drinks in Asia and pine needles have been used in traditional medicine for liver diseases, skin diseases, and hypertension [13,14]. Essential oils constitute of the most important group of pharmacologically active components of the genus *Pinus* [15,16,17]. Essential oils, volatile products of a plant’s secondary metabolism, possess well-known antioxidant properties [5,6,7,8,9]. The needles of the genus *Pinus* contain essential oils and the components of their essential oils have been established through chromatographic techniques [17,18,19]. However, most of these efforts used different experimental conditions and there is little comprehensive information on the volatile compounds and biological activities of the essential oils of pine needles.

In the present study the essential oils of needles from six of the *Pinus* taxa endemic to China (including *P. tabulaeformis*, *P. tabulaeformis* f. shekanensis, *P. tabulaeformis* var. mukdensis, *P. tabulaeformis* var. umbraculifera, *P. henryi* and *P. massoniana*) were obtained by steam distillation. Their chemical components were separated and identified by gas chromatography/mass spectrometry (GC/MS). In addition, total phenolic content, DPPH free radical scavenging activity, ferric reducing antioxidant power (FRAP) and ABTS radical cation scavenging activity assays were applied to accurately evaluate the antioxidant properties of the oils. Hopefully, this study will provide sufficient experimental evidence of antioxidant activity and potential for further development and utilization of these *Pinus* taxa.

## 2. Results and Discussion

### 2.1. Essential Oil Composition

Clear yellow volatile oils were obtained by hydrodistillation of needles from *P*. *tabulaeformis*, *P*. *tabulaeformis* f. *shekanensis*, *P*. *tabulaeformis* var. *mukdensis*, *P*. *tabulaeformis* var. *umbraculifera*, *P*. *henryi* and *P*. *massoniana* at 0.51%, 0.50%, 0.47%, 0.42%, 0.48% and 0.53% yield, respectively (Table 1). Bo *et al.* observed that the yield of essential oil from *P*. *massoniana* and *P*. *sylvestris* was 0.50% and 0.43% [20], while Zafar *et al.* reported that the yield of essential oil from *P*. *roxburghaii* needles was 0.11% [21]. The content of essential oil is influenced by pretreatment of the leaves, ratio of water and leaves, extraction time and collection season [22].

The chemical composition of the essential oils from the six *Pinus* taxa was analysed by GC-MS. Qualitative and quantitative analytical results are shown in Table 1. In total, 72 constituents were identified in the essential oils, while 57 (representing 93.76% of the total amount) were found in *P*. *tabulaeformis*; 47 (representing 95.03% of the total amount) in *P*. *tabulaeformis* f. *shekanensis*; 46 (representing 95.93% of the total amount) in *P*. *tabulaeformis* var. *mukdensis*; 23 (representing 95.00% of the total amount) in *P*. *tabulaeformis* var. *umbraculifera*; 61 (representing 95.77% of the total amount) in *P*. *henryi* and 43 (representing 97.03% of the total amount) in *P*. *massoniana*. β-Caryophellene and α-pinene were the main constituents of all oils; this has also been reported elsewhere [23,24]. The contents of β-caryophellene for *P*. *tabulaeformis*, *P*. *tabulaeformis* f. *shekanensis*, *P*. *tabulaeformis* var. *mukdensis*, *P*. *tabulaeformis* var. *umbraculifera*, *P*. *henryi* and *P*. *massoniana* were 22.36%, 20.83%, 24.08%, 26.31%, 18.26% and 18.48%, respectively, and the contents of α-pinene were 11.08%, 6.78%, 16.55%, 20.55%, 9.68% and 7.81%, respectively. For germacrene D the values were 7.43% (*P*. *tabulaeformis*), 8.97% (*P*. *tabulaeformis* f. *shekanensis*), 9.93 (*P*. *tabulaeformis* var. *mukdensis*) and 9.78% (*P*. *massoniana*). Other major compounds were bornyl acetale (12.71%), α-guaiene (8.19%) in *P*. *tabulaeformis* var. *umbraculifera* and γ-cadinene (9.74%) for *P*. *tabulaeformis* f. *shekanensis*. Based on the results, the essential oils of the six *Pinus* taxa have different chemical compositions in terms of major constituents. Aside from the genetic variation, some reports have emphasized the influence of the age of the plant, the harvest period, the climate and the geographic circumstances on the components of the essential oil [17,25], which explains the chemical composition differences among the six *Pinus* taxa.

**Table 1:** Chemical composition of the essential oils of the six *Pinus* taxa.

No.	Name of Components	RI	Relative Peak Area (%)							
			Pt	Ptf	Pvm	Pvu		Ph		Pm	
1	Tricyclene	921	1.81	1.11	0.39	-		0.21		-
2	α-Thujene	925	0.13	1.48	0.24	-		1.72		2.02
3	α-Pinene	932	11.08	6.78	16.55	20.55		9.68		8.16
4	Camphene	945	0.38	0.45	0.58	-		0.34		0.63
5	Sabinene	970	0.15	-	-	-		0.18		-
6	β-Pinene	978	2.29	0.95	1.45	-		0.35		2.99
7	Myrcene	982	-	-	0.72	-		1.04		-
8	α-Phellandrene	1003	0.26	-	-	-		-		-
9	δ-3-Carene	1008	0.31	0.19	0.38	-		0.13		0.13
10	α-Terpinene	1014	-	-	0.11	-		0.12		0.29
11	Limonene	1024	0.41	-	-	-		0.09		-
12	β-Phellandrene	1027	0.12	0.30	0.57	-		0.37		0.66
13	β-Ocimene	1041	-	-	0.22	-		0.15		-
14	γ-Terpinene	4056	0.48	0.22	0.33	-		0.13		0.52
15	α-Terpinolene	4086	0.24	0.37	0.95	-		0.59		0.17
16	Terpinen-4-ol	1161	-	-	-	-		0.15		-
17	α-Terpineol	1175	3.43	2.62	3.32	-		1.32		3.34
18	Bornyl acetale	1267	4.13	3.32	3.93	12.71		2.96		3.83
19	α-Cubebene	1346	0.21	0.32	0.28		-	0.26		0.24		
20	α-Rlangene	1371	0.24	0.26	0.30		-	0.31		0.24		
21	α-Copaene	1379	-	-	0.20		-	-		0.14		
22	β-Bourbonene	1388	-	-	-		1.13	0.12		-		
23	β-Elemene	1391	1.26	2.13	1.82		-	1.41		2.93		
24	Longifolene	1411	0.54	0.29	0.12		1.52	0.24		-		
25	β-Caryophellene	1421	22.36	20.83	24.08		26.31	18.26		18.48		
26	α-Caryophellene	1423	3.72	-	-		-	2.64		3.36		
27	α-Guaiene	1437	2.80	2.07	1.23		8.19	2.48		3.67		
28	Aromadendrene	1440	2.15	3.18	3.54		-	2.22		2.31		
29	(*E*)-β-Farnesene	1456	1.45	0.77	-		1.25	2.59		-		
30	β-Santalene	1458	0.47	-	0.83		1.27	-		-		
31	α-Humulene	1461	-	-	2.65		-	0.85		-		
32	γ-Muurolene	1472	1.06	-	-		2.30	-		-		
33	Germacrene D	1482	7.43	8.97	9.93		1.26	2.71		9.78		
34	α-Amorphene	1484	0.42	0.86	0.60		0.27	0.72		0.80		
35	Aristolochene	1486	2.10	3.56	2.95		-	3.03		3.71		
36	β-Selinene	1488	1.60	-	0.22		1.01	1.23		-		
37	Phenylethyl-3 methyl butanoate	1490	0.41	0.47	-		1.18	0.27		-		
38	Phenylethyl isovalerate	1491	0.74	1.69	1.49		-	0.14		1.23		
39	*epi*-Cubebol	1495	0.44	0.24	-		1.37	1.17		-		
40	α-Selinene	1496	1.72	2.42	2.34		-	3.53		2.91		
41	Bicyclogermacrene	1498	-	0.35	-		0.83	2.97		-		
42	α-Muurolene	1500	0.62	-	-		1.79	-		-		
43	β-Cadinene	1508	2.00	-	1.07		-	-		0.92		
44	γ-Cadinene	1517	3.72	9.74	2.19		3.45	2.23		1.62		
45	δ-Cadinene	1521	2.33	1.31	-		-	2.57		-		
46	Cadina-1,4-diene	1525	-	-	-		-	0.12		1.55		
47	α-Cadinene	1533	-	-	-		-	0.19		1.36		
48	α-Calacorene	1542	0.61	0.16	0.35		-	0.11		0.51		
49	(*E*)-Nerolidol	1546	0.24	1.31	0.35		-	1.17		2.50		
50	Occidentalol	1553	-	-	-		-	2.33		-		
51	Germacrene B	1557	0.20	1.50	0.81		-	0.22		0.12		
52	(*Z*)-3-Hexenyl benzoate	1567	0.16	0.18	0.58		-	0.71		1.22		
53	Longipinaol	1573	-	0.15	-		-	1.77		0.64		
54	Spathulenol	1579	0.23	-	0.60		1.25	0.65		-		
55	Caryophyllene oxide	1585	0.30	3.59	1.16		-	-		1.74		
56	Globulol	1593	0.15	0.45	1.40		-	2.62		1.18		
57	β-Calacorene	1596	2.25	1.40	2.44		3.64	1.28		-		
58	β-Oplopenone	1608	0.49	2.87	0.81		-	0.18		1.91		
59	δ-Cadinol	1646	-	0.44	0.61		-	2.98		0.91		
60	α-Cadinol	1652	-	-	-		1.55	-		-		
61	α-Eudesmol	1654	0.17	0.74	-		-	1.74		1.76		
62	β-Bisabolal	1672	0.15	-	-	-		0.76	1.98		
63	Pentadecanal	1718	0.17	0.18	-	-		0.48	1.21		
64	Benzyl benzoate	1733	1.10	0.90	0.69	-		-	-		
65	Octadecane	1798	0.23	2.07	-	-		2.54	-		
66	Cubitene	1877	0.56	-	0.29	0.48		-	-		
67	Laurenene	1881	0.52	-	-	-		0.36	-		
68	Pimaradiene	1943	0.23	0.62	-	-		0.47	-		
69	Neocembrene	1960	0.14	0.11	-	1.69		1.87	0.18		
70	Sclareol	1973	0.28	0.89	0.26	-		1.38	-		
71	Manoyl oxide	1993	0.57	-	-	-		-	-		
72	Palustradiene	2005	-	0.22	-	-		0.36	2.18		
	Total percentage ^a^		0.51	0.50	0.47	0.42		0.53	0.48		
	Essential oil (%) content		93.76	95.03	95.93	95.00		95.77	97.03		

RI: retention index according to n-hydrocarbons (C_6_–C_22_) on the HP-5MS column; Pt: *P*. *tabulaeformis*; Ptf: *P*. *tabulaeformis* f. *shekanensis*; Pvm: *P*. *tabulaeformis* var. *mukdensis*; Pvu: *P*. *tabulaeformis* var. *umbraculifera*; Ph: *P*. *henryi*; Pm: *P*. *massoniana*; ^a^ Percentage of the total peak area. Components with percentage ≥ 0.1% are presented; -: not detected.

### 2.2. Total Phenolic Content

**Figure 1:**
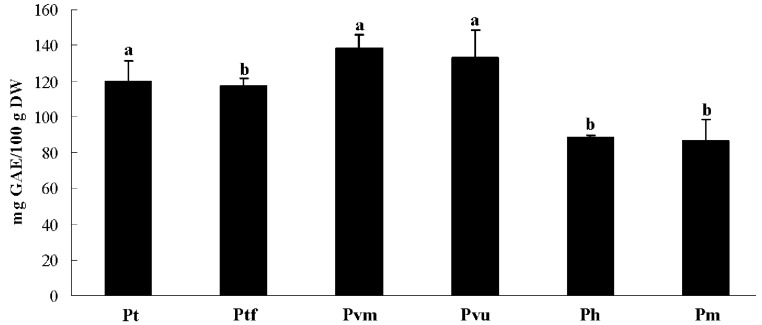
Total phenolics content of six *Pinus* tax extracts. Means with different letters indicate significant differences at the 0.05 level. Taxon codes are identified in Table 1.

Phenolic compounds are often correlated to the antioxidant activity due to their capability to act as electron donors in free radical reactions [26]. In this study, total phenolic content (TPC) was estimated by the Folin-Ciocalteu colorimetric method using gallic acid as standard. The TPCs of the extracts ranged from 86.60 to 138.34 mg GAE/100 g DW, with *P*. *tabulaeformis* var. *mukdensis* showing the highest value of 138.34 mg GAE/100 g DW, followed by *P*. *tabulaeformis* var. *umbraculifera*, *P*. *tabulaeformis*, *P*. *tabulaeformis* f. *shekanensis*, *P*. *henryi* and *P*. *massoniana* with TPC values of 133.35, 119.68, 117.08, 88.61 and 86.60 mg GAE/100 g DW, respectively (Figure 1). Generally, aside from genetic differences, it has been shown that temperature and altitude are one of the major environmental factors that affect plant composition and properties [27,28,29]. Wildi and Lutz found that the plant samples collected from lower temperature sites showed higher phenolic content. In this work, *P*. *tabulaeformis* var. *mukdensis* and *P*. *tabulaeformis* var. *umbraculifera* were collected from a lower temperature site (Liaoning Province) compared to the other four taxa (Shaanxi Province). In addition to genetic differences, this might be one of the reasons why *P*. *tabulaeformis* var. *mukdensis* and *P*. *tabulaeformis* var. *umbraculifera* had higher phenolic content than the other taxa.

### 2.3. Antioxidant Activity

Antioxidant activity is a complex process usually occurring through several mechanisms. The evaluation of the antioxidant activity should be carried out by more than one test method [30]. In this study, three antioxidant assays, DPPH free radical scavenging activity, ferric reducing antioxidant power (FRAP) and ABTS radical cation scavenging activity were applied to accurately evaluate the antioxidant properties of the six *Pinus* taxa. The three assays gave very different values in absolute terms (*i.e.*, μmol trolox equivalent (TE)/g DW), but showed the same relative pattern. Among taxa, the highest antioxidant activity was observed in *P. tabulaeformis* var. *Mukdensis*, regardless of the assay method used.

The antioxidant activity of all extracts determined by DPPH radical scavenging ability ranged from 892.45 to 1851.65 μmol TE/g DW. The highest antioxidant activity was found in the needle extract of *P.* *tabulaeformis* var. *mukdensis*, followed by *P*. *tabulaeformis* f. *shekanensis*, *P*. *tabulaeformis* var. *umbraculifera*, *P*. *tabulaeformis*, *P.* *massoniana* and *P*. *henryi* (Table 2). The greatest ferric reducing antioxidant power of 1134.45 μmol TE/g DW was detected in *P. tabulaeformis var. mukdensis*, while the *P*. *massoniana* had the lowest power of 477.78 μmol TE/g DW. Based on the mean value of each taxon, the ferric ion reducing power rank was *P.* *tabulaeformis* var. mukdensi*s* > *P*. *tabulaeformis* > *P*. *tabulaeformis* f. *shekanensis* > *P*. *tabulaeformis* var. *umbraculifera* > *P*. *henryi* > *P*. *massoniana*. The ferric reducing antioxidant power of all taxa listed in Table 2 shows a different order from the DPPH assay results. These differences could be attributed to the different stoichiometry of the DPPH and FRAP assay reactions. In addition, the compositional differences in extracts and their different solubility in the test systems may also affect their antioxidant activity [31,32]. In the ABTS radical cation scavenging activity assay (Table 2), the rank order based on the average TE values was as follows: *P. tabulaeformis* var. *mukdensis* (3584.41 μmol TE/g DW) > *P*. *tabulaeformis* var. *umbraculifera* > *P*. *tabulaeformis* > *P*. *tabulaeformis* f. *shekanensis* > *P*. *henryi* > *P*. *Massoniana* (1461.01 μmol TE/g DW). 

The results of this study showed that the aqueous extracts of the six *Pinus* taxa had strong antioxidant activity, including DPPH radical, ferric reducing antioxidant power (FRAP) and ABTS radical cation scavenging capacity. Among the tested *Pinus* taxa, *P*. *tabulaeformis* var. *mukdensis* had the greatest antioxidant activities, while *P*. *massoniana* displayed the lowest antioxidant capacities. Compared with some popular plant species, the six tested *Pinus* taxa had higher antioxidant capacities than *Phyllostachys pubescens* [33], *Ascophyllum nodosum* [34], *Sorghum bicolor* [35], *Vaccinium corymbosum* [36] and *Phaseolus vulgarisbut* [37], but lower than some other popular plant species, such as *Camellia sinensis* [6] and *Oenocarpus bacaba* [38], as measured by their radical cation scavenging activity. Pine needles have been used to prepare drinks in Eastern Asia and as a traditional medicine for several centuries in China [13,14], thus the results in this study indicate that pine needles could be a potential source of natural antioxidant foods and also provide data to health professionals and food policy makers for encouraging the population to consume these plants.

**Table 2:** Antioxidant activity determined by the DPPH, FRAP and ABTS assays of the needles extracts from the six *Pinus* taxa.

Taxa	DPPH	FRAP	ABTS
Pt	1775.22 ± 138.17 ^NF^	1036.68 ± 51.14 ^a^	3078.52 ± 278.59 ^a^
Ptf	1844.19 ± 180.55	904.72 ± 90.73 ^b^	2467.85 ± 141.63 ^b^
Pvm	1851.65 ± 151.19	1134.45 ± 36.14 ^ab^	3584.41 ± 315.63 ^a^
Pvu	1817.25 ± 131.19	814.72 ± 112.41 ^b^	3486.33 ± 140.75 ^c^
Ph	918.28 ± 25.37	584.78 ± 68.67 ^c^	2151.43 ± 215.03 ^b^
Pm	892.45 ± 78.31	477.78 ± 48.67 ^c^	1461.01 ± 131.03 ^c^

All values are mean ± SD; ^a–c^ Values with different letters in the same column were significantly (*p* < 0.05) different; NF: not significant. Taxon codes are identified in Table 1.

### 2.4. Correlation Analysis

Correlation analysis was used to explore the relationships amongst the different antioxidant variables measured for extracts of the six *Pinus* taxa (Table 3). Regarding the different methods, a significant correlation between methods was confirmed with the three methods (DPPH, FRAP and ABTS). The correlation coefficient was 0.90 between DPPH and FRAP, and 0.84 between DPPH and ABTS at 0.05 level. Moreover, the results of TPC in the studied taxa correlated significantly and positively with their antioxidant capacity determined by DPPH (*r* = 0.94), FRAP (*r* = 0.93) and ABTS (*r* = 0.86) methods at 0.01 level. This was in agreement with earlier reports which stated that plants with higher content of polyphenolic compounds possess stronger antioxidant activity [39,40,41]. Therefore, the total phenolics assay could be a suitable candidate for measuring the antioxidant capacity of the six *Pinus* taxa.

**Table 3:** Linear correlation coefficients between phenolic content and antioxidant capacity (panel A), and among the different methods for quantifying antioxidant capacity (panel B).

	DPPH	FRAP	ABTS
***Panel A***			
TPC	0.94 **	0.93 **	0.86 *
***Panel B***			
DPPH	1.00		
FRAP	0.90 *	1.00	
ABTS	0.84 *	0.63	1.00

* Correlation is significant at the 0.05 level. ** Correlation is significant at the 0.01 level.

## 3. Experimental Section

### 3.1. Chemicals and Plant Materials

2,2-Diphenyl-1-picrylhydrazyl (DPPH) was obtained from Tokyo Chemical Co. (Tokyo, Japan). 2,4,6-Tripyridyl-s-triazine (TPTZ), 6-hydroxy-2,5,7,8-tetram-ethylchroman-2-carboxylic acid (Trolox), 2,2′-azinobis-(3-ethylbenzothiazoline-6-sulfonic acid) diammonium salt (ABTS), gallic acid, rutin, Folin-Ciocalteu reagent and a mixture of aliphatic hydrocarbons (C_6_–C_22_) were from Sigma Chemical Co. (St. Louis, MO, USA). All the other chemicals used were analytical grade.

The needles of *P*. *tabulaeformis*, *P*. *tabulaeformis* f. *shekanensis*, *P*. *tabulaeformis* var. *mukdensis*, *P*. *tabulaeformis* var. *umbraculifera*, *P*. *henryi* and *P*. *massoniana* were collected from different regions in China (Table 4). The samples were dried in a shady ventilated place, then cut into 1 cm pieces and separately hydrodistilled for 4 h (200 g sample in 500 mL of distilled water) in a Clevenger-type apparatus with a water cooled receiver, in order to reduce hydrodistillation overheating artifacts. The essential oil was taken up in diethyl ether, dried over sodium sulphate and reduced in volume at room temperature under vacuum on a rotatory evaporator. The oil obtained was stored at 4 °C prior to studies. The yields of the essential oils were calculated by the formula:

$$\text{Yield of essential oil}=\frac{\text{Volume of essential oil (g)}}{\text{Volume of sample (g)}}\times 100\%$$

**Table 4:** The origins of the six *Pinus* taxa with their geographical characteristics.

Taxa	Origin	Latitude(°N)/Longitude(°E)	Elevation (m)
*P. tabulaeformis*	Huanglong, Shaanxi	35.632/109.772	1127
*P. tabulaeformis* f. *shekanensis*	Fuxian, Shaanxi	35.998/108.690	1316
*P. tabulaeformis* var. *mukdensis*	Anshan, Liaoning	40.960/123.147	294
*P. tabulaeformis* var. *umbraculifera*	Anshan, Liaoning	41.009/123.124	250
*P. massoniana*	Yangxian, Shaanxi	33.326/107.624	722
*P. henryi*	Nanzheng, Shaanxi	32.857/106.586	1254

### 3.2. Identification of the Chemical Components of the Essential Oils

The GC/MS analysis was carried out using splitless injection mode on a ULTR-Polaris Q GC-MS instrument (Thermo Electron Corporation, Waltham, MA, USA), equipped with a HP-5MS capillary column (30 m × 0.32 mm, 0.25 μm film thicknesses). Helium was used as carrier gas at a flow rate of 1 mL/min in the split mode (split ratio 1:50), with an injection vol. 0.2 µL. Oven temperature was programmed from 50 °C (3 min) to 260 °C (5 min) at 2 °C/min and injector heater 250 °C. The mass-spectrometer was operating (full scan-mode) in the EI-mode at 70 eV. The components of essential oils were identified by matching their recorded mass spectra with the data bank mass spectra (NIST 98, NIST 02, NIST 05, and NIST 08) and by comparing their retention indices relative to a series of n-hydrocarbons (C_6_–C_22_) with literature values [42]. For each compound on the gas chromatogram, the percentage of peak area relative to the total peak area of all compounds was determined and reported as relative amount of that compound, without using correction factors.

### 3.3. Determination of Total Phenolic

The total phenolic content (TPC) was determined using Folin-Ciocalteu method [43]. In this method, extract (0.1 mL, 1 mg/mL) was reacted with Folin-Ciocalteu reagent (0.1 mL) and then neutralized with sodium carbonate (10 mL, 7%, v/v; in distilled water). After 60 min of incubation, the absorbance of the solution was measured at 765 nm. The results were expressed as the equivalent to milligrams of gallic acid per 100 gram of dry weight (mg GAE/100 g).

### 3.4. Antioxidant Capacity Determined by DPPH

The ability to scavenge DPPH free radicals was determined based on the method of Brand-Williams *et al.* with minor modifications [44]. Briefly, 0.1 mM DPPH solution (190 μL) was mixed with each test sample solution (10 μL) in 96-well plates. After the reaction was allowed to take place in the dark for 30 min, the absorbance at 517 nm was recorded to determine the concentration of remaining DPPH. Results were expressed as trolox equivalent antioxidant capacity.

### 3.5. Antioxidant Capacity Determined by Ferric Reducing Antioxidant Power (FRAP)

The FRAP assay was determined according to Benzie and Strain with some modifications [45]. FRAP reagent consist of 10 mM TPTZ in 40 mM HCl, 20 mM ferric chloride and 300 mM acetate buffer (pH 3.6) in the ratio of 1:1:10 (v/v/v). For this assay, FRAP reagent (300 μL) was mixed at 37 °C with each test sample solution (10 μL) in 96-well plates. After 10 min, the coloured products were then taken at 593 nm. Results were expressed as trolox equivalent antioxidant capacity.

### 3.6. Antioxidant Capacity Determined by Radical Cation (ABTS)

The ABTS assay was based on the method of Re *et al.* with slight modifications [46]. ABTS radical cation (ABTS^+^) was produced by reacting 7 mM ABTS solution with 2.45 mM potassium persulphate and allowing the mixture to stand in the dark at room temperature for 12–16 h before use. The ABTS^+^ solution was diluted with ethanol to an absorbance of 0.70 ± 0.02 at 734 nm. For this assay, ABTS radical solution (190 μL) was mixed with each test sample solution (10 μL) in 96-well plates. After 6 min, the decrease of absorbance was measured at 734 nm using a micro plate reader. Results were expressed as trolox equivalent antioxidant capacity.

### 3.7. Statistical Analysis

All analyses were performed in triplicate and the results expressed as the mean ± standard deviation (SD). Data analysis was carried out by one-way ANOVA followed by Tukey’s post hoc test at *p* < 0.05 using the SPSS version 18.0 for Windows. A two-tailed Pearson’s correlation test was processed to determine the correlations among means.

## 4. Conclusions

This paper describes a comparative study of the chemical composition and antioxidant activity of essential oils from needles of the six *Pinus* taxa endemic to China. In light of the results obtained, we can conclude that there are obvious differences in the relative contents of chemical constituents among the six *Pinus* taxa’ essential oils, although their main constituents were similar and differed slightly in amount. Antioxidant evaluation (DPPH free radical scavenging activity, ferric reducing antioxidant power (FRAP) and ABTS radical cation scavenging activity assays) of the extracts showed that the antioxidant potency correlated well with the total phenolic content (TPC) and revealed that all essential oils exhibit acceptable antioxidant activities, which strongly suggests that these pine needles may serve as a potential source of natural antioxidants for food and medical purposes.
